# A Recipe for Self-Renewing Brain

**DOI:** 10.1371/journal.pbio.0030307

**Published:** 2005-08-16

In all the hullabaloo about stem cells, nobody has noted their uncanny similarity to pizza dough. You can divide either into two or four or eight identical pieces, but that doesn't determine what kind of cell or pizza you're going to make. But once you let a cell grow hundreds of nuclei, or you pile on the pepperoni, you're on your way to making a skeletal muscle fiber or a pepperoni pizza. If you want a white blood cell or an all-veggie pie, you're out of luck. The commitment to becoming a certain cell type is called differentiation.

Stem cells in living organisms can multiply without differentiating, preserved by molecular signals in special niche environments; without these signals in the petri dish, they differentiate. Pluripotent mouse embryonic stem (ES) cells, a special type of stem cell with the potential to develop into many different cell types, are an exception. Because they divide symmetrically, the scads of artificially grown ES cells are all the same. This leads researchers to wonder: what conditions in the body keep stem cells from differentiating, why are ES cells the only kinds that don't differentiate in the petri dish, and how can scientists create undifferentiated tissue-specific stem cells in the lab?

In a new paper, Austin Smith and colleagues developed a method to produce symmetrical divisions of mouse brain stem cells derived from ES cells. Their novel method creates an on/off switch for differentiation of tissue-specific stem cells: they can multiply without differentiation, and they can also become normal brain cells. The authors also managed to cultivate the brain stem cells without re-creating the rarefied neurosphere, the highly specialized environment or microenvironment in which the body grows its own brain stem cells.

Many scientists believe that in the body, these microenvironments prevent stem cells from differentiating. Neurospheres, for example, contain some undifferentiated brain stem cells floating in a broth of differentiating cells. One feature of the neurosphere is that a very low percent of cells are brain stem cells. In fact, neurospheres have so few of these cells that scientists have a hard time even observing them. But by cultivating brain stem cells outside the neurosphere, the scientists showed that a complex microenvironment may not be necessary. To grow their stem cells, Smith et al. combined epidermal growth factor (EGF) and fibroblast growth factor (FGF), two small proteins that bind to stem cells and promote growth. [Fig pbio-0030307-g001]

**Figure pbio-0030307-g001:**
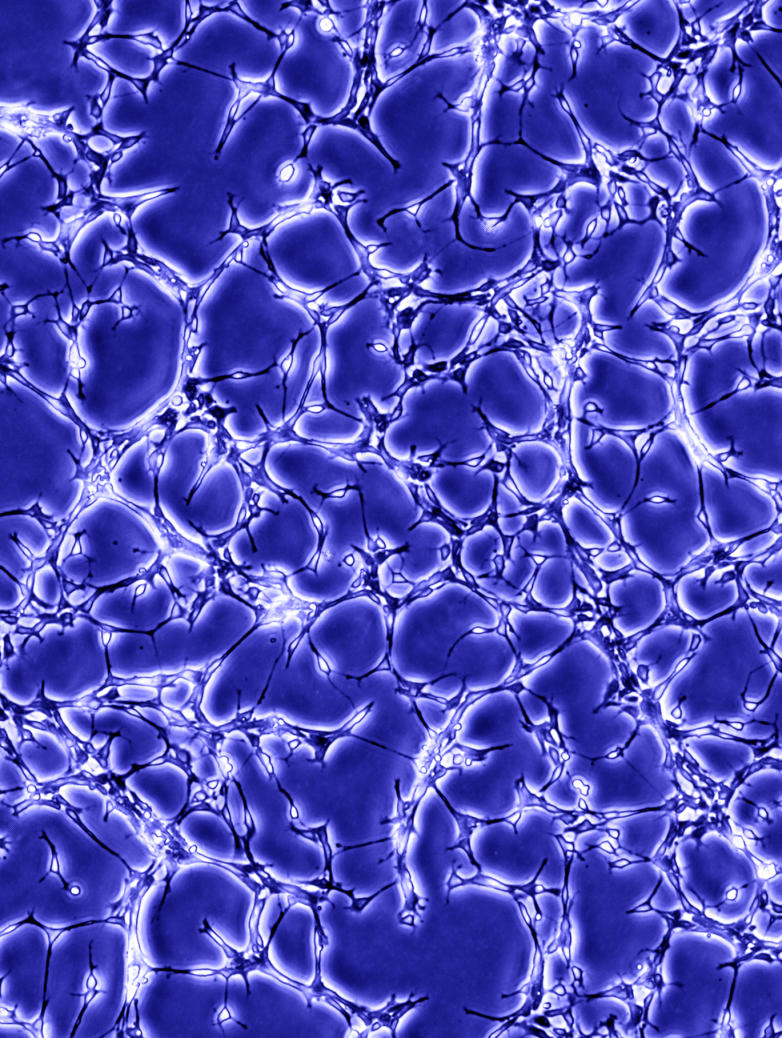
A new technique allows researchers to culture brain stem cells in unlimited numbers and then induce differentiation into mature neurons or glia by changing growth factors

Previously, scientists had grown brain stem cells with FGF. Upon removing FGF, the cells failed to differentiate and become mature. The cells that Smith et al. grew, in contrast, became mature cells upon removal of the growth factor cocktail. They observed both neurons and astrocytes, the two types of cells into which the brain stem cells mature.

In the future, scientists may use this new technique to produce large quantities of the cells to study their basic properties and also to explore their value for modeling neurodegenerative afflictions, including Huntington disease, Parkinson disease, and Alzheimer disease. Additionally, these cells may clinch the debate of whether doctors will be able to use stem cells directly to repair brain damage.

